# Preoperative diagnostic value of multimodal spectral CT for patients with atrial fibrillation undergoing radiofrequency ablation

**DOI:** 10.3389/fmed.2024.1440020

**Published:** 2024-09-12

**Authors:** Na Yu, Yuqin Hong, Xue Lv, Qiao Liu, Min Yan

**Affiliations:** Department of Radiology, The Third Affiliated Hospital of Chongqing Medical University, Chongqing, China

**Keywords:** atrial fibrillation, spectral CT, delayed enhancement, LAA thrombus, iodine concentration, left atrium and pulmonary vein

## Abstract

**Objective:**

Delayed enhancement cardiac computed tomography (CT) empowers the diagnosis of left atrial appendage thrombus while limited to scanning heterogeneity. We optimized the spectral CT scan and post-process protocols, incorporating delayed enhancement and spectral iodine analysis to discriminate left atrial appendage (LAA) thrombus with better morphological relationships between the left atrium, pulmonary vein, and esophagus.

**Methods:**

A total of 278 consecutive patients were retrieved from January 2019 to June 2023. All patients underwent transesophageal echocardiography (TEE) and spectral CT scan of the left atrial and pulmonary vein, with a complete period including the pulmonary venous phase and three delay phases. TEE diagnosis was used as the standard reference. For patients exhibiting LAA filling defects during the pulmonary venous phase, a delayed scan of 30 s (phase I) was performed. If the filling defects persisted, a further delayed scan of 1 min (phase II) was conducted. In cases where the filling defects persisted, an additional delayed scan of 2 min (phase III) was carried out. Iodine concentration in the filled defect area of LAA and the left atrium was measured in phase III. Moreover, 30 patients were randomly selected for water-swallowing and the other 30 for calm breathing. The image quality and esophageal dilation of the two groups were assessed by two experienced surgeons specializing in radiofrequency ablation.

**Results:**

In total, 14 patients were diagnosed with thrombi by TEE. The sensitivity, specificity, positive predictive values, negative predictive values, and AUC of phase III delayed combined with iodine quantification for thrombi diagnosis were all 100%. The water-swallowing group exhibited significantly greater esophageal filling and expansion than the calm-breathing group, contributing to a better morphology assessment with no significant difference in image quality.

**Conclusion:**

Combined with iodine quantification, delayed enhancement of spectral CT imaging presents a promising diagnostic potency for LAA thrombus. Incorporating water swallowing into the CT scan process further enables anatomical visualization of the esophagus, left atrium, and pulmonary vein, thereby providing more objective and authentic imaging evidence to assess the esophageal morphology and positional relationships.

## Introduction

Atrial fibrillation (AF) is one of the most common heart rhythm disorders, which has impacts on cardiovascular morbidity and leads to heart failure and other life-quality reductions, increasingly endangering life and health (1, 2). Radiofrequency ablation of pulmonary vein isolation is a popular method to treat atrial fibrillation (3, 4). Before radiofrequency ablation, a preoperative examination is indispensable to determine the anatomic position of the left atrium, pulmonary vein, and esophagus, and to rule out the presence of thrombus in the left atrial appendage (LAA). The anatomical relation was evaluated by a CT scan and image reconstruction, while the LAA thrombus was identified by gold-standard transesophageal echocardiography (TEE). However, TEE is a semi-invasive examination with an incidence of esophageal injury of approximately 30% (3), posing challenges to patient cooperation.

Enhanced CT is a safe and non-invasive imaging technique; however, due to the unique anatomical structure and circulation deposition of LAA, filling defects of iodine contrast agent may occur during routine early enhancement, leading to false positives and reduced specificity for thrombus diagnosis. Previous studies have demonstrated that additional delayed enhanced phases can enhance the diagnostic accuracy of LAA thrombus. However, the selection of delay time is primarily reliant on operator experience, leading to significant subjectivity ranging from 30 s to 6 min and resulting in variations in positive predictive value (PPV) and specificity (5–10). International guidelines still regard TEE as the gold standard for LAA thrombus diagnosis, suggesting that more evaluations are necessary for CT as a replacement for TEE in the thrombus diagnosis (11, 12). Here, our study incorporated spectral iodine concentration into the delayed enhancement of the CT scan, and we quantified the iodine concentration of the LAA during the delay period. Simultaneously, we further optimized the scan and post-process protocol for the pulmonary venous phase. Before image acquisition, patients were instructed to swallow distilled water to expand the esophagus, thereby facilitating a more precise visualization of the anatomical relationship between the left atrium, pulmonary vein, and esophagus. Our research develops an optimal CT scan and post-process protocol to acquire multimodal image data to elucidate the anatomical relationship with enhanced accuracy in diagnosing LAA thrombus. The ultimate goal is to replace TEE with CT to reduce unnecessary invasive examination and surgical risk.

## Method

### Study populations

The study was approved by the Ethics Committee of the Third Affiliated Hospital of Chongqing Medical University (Chongqing, China). A total of 278 consecutive patients (181 men (65%), mean age of 56 ± 13 years, range 32–81 years) were prospectively enrolled from January 2019 to June 2023. The inclusion and exclusion criteria of patients were as follows; inclusion criteria: age ≥ 18 years, clinical diagnosis of non-valvular atrial fibrillation, and underwent both TEE and spectral CT-scan of the pulmonary veins phase and three delayed phases before catheter radiofrequency ablation; exclusion criteria: renal insufficiency (glomerular filtration rate < 30 mL/min); iodine contrast agent allergy; poor image quality. All patients had signed the informed consent to admission and met the ethical requirements. All patients were included in an electronic database, all clinical and imaging features were recorded, and the CHADS _2_ score was calculated.

### CTPV protocol

Preparation before CT examination: patients were given detailed introductions to the examination process and possible discomfort reactions to the iodine contrast agent to eliminate their nervousness and achieve better cooperation. Thirty of the 278 patients were randomly selected to wrap a mouthful of purified water before iodine contrast injection and image acquisition, and instructed to swallow water when image acquisition was performed to fully dilate the esophagus.

All patients underwent 256-slice spectral CT (Revolution, GE Healthcare) scanning with prospective electrocardiogram (ECG) gating. A nonionic, iso-osmolar contrast material (iohexol, 350 mg of iodine per ml, Omnipaque 350; General Electric Healthcare) was administered intravenously at the dose of 80–100 mL, followed by 30 mL of saline solution at a rate of 4.5 mL/s by using a dual-shot injector (Nemoto Kyorindo). The region of interest (ROI) was placed in the left atrium, dynamically monitored the CT value, and the scan was automatically triggered when it reached 150 HU. Simultaneously, the water-swallowing group was instructed to swallow water calm-breathing group maintained calm breathing throughout the scan. The pulmonary venous phase scan range was from thoracic inlet to bilateral costophrenic Angle. The range of delayed enhancement scans only covered the LAA to reduce unnecessary radiation doses. If filling defects were observed in the LAA during the pulmonary venous phase, an image was acquitted after a 30-s delay (phase I delay). In case the filling defects persisted, another image was acquitted after a 1-min delay (phase II delay). If the filling defects persisted, an additional image was captured following a 2-min delay (phase III delay). A spiral scan was performed with pitch 0.992, detector width 80 mm, rotation speed 0.5 s, tube voltage 100–120 kV, automatic mA tube current, noise index 22.0, and reconstruction slice thickness 0.625 mm. The adaptive statistical iterative reconstruction (ASIR, GE) algorithm (50% filter back-projection blend) was used for reconstruction to reduce radiation dose without affecting the overall image quality (13). The CT scan length, time, and dose length product (DLP) are recorded from the scanner console, and the effective radiation dose at different phases is calculated by multiplying the DLP with the previously recommended conversion factor of 0.014 mSv/cGy/cm (14).

### Multimodal image post-processing and analysis

The raw data were loaded to AW 4.7 post-process workstation, and reconstructed to three-dimensional anatomical structures of left atrial and pulmonary vein and esophagus. The esophagus, being a hollow tube, exhibits a significantly distinct CT value compared to the left atrium injected with the iodinated contrast agent, and needs a separate reconstruction. The left atrium, pulmonary vein, and esophagus were fused by fusion imaging technology, and the esophagus was stained blue to visually show the positional relationship of the three. The reconstruction image quality of the water-swallowing group and the calm-breathing group was evaluated by the radiofrequency ablation surgeon, including background noise and overall image quality. The overall image quality evaluation includes the large vascular structure at the left atrial level, mediastinal structure, and distal small vessels. The 5-point Likert scale was used to score normal and abnormal tissues. The average CT value of the ascending aorta in the left atrial plane, and the CT value and standard deviation (SD) of the back muscles in the same plane were measured in the two groups. The image signal-to-noise ratio (SNR) and contrast-to-noise ratio (CNR) were calculated as follows: SNR = CT _aorta_/SD _muscle_; CNR = (CT _aorta_-CT _muscle_)/SD _muscle_. The iodine concentration of the LAA and left atrium at the same level was measured from the reconstructed iodine map of phase III delayed spectral images.

### Transesophageal echocardiography

**Figure 1:**
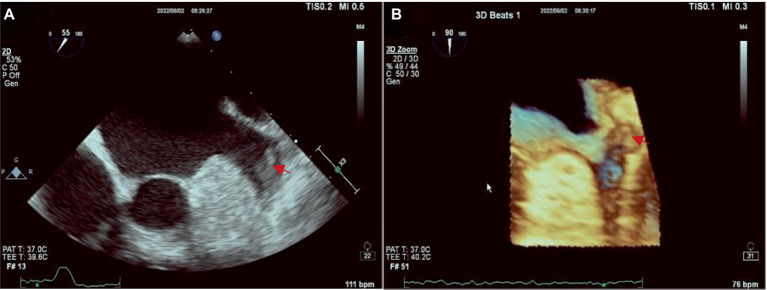
**(A)** The tip of LAA was a fresh thrombus (red arrow) at TEE. **(B)** Image of 3D ultrasound.

The Philips APQ7C ultrasound device was used for TEE examination in this study. The TEE examination of LAA involves placing a probe into the esophagus and stomach and scanning the heart from back to front after the patient has taken an anesthetic. At this moment, the probe is closest to the heart base and atrium, and TEE can easily capture clear images of the LAA at 45, 90, and 135 degrees of each standard section, to observe whether the hypoechoic thrombus attachment in the left auricular wall and apex of auricular. Three-dimensional reconstruction of the left auricle can also be performed to understand its morphological and structural characteristics. Additionally, pulse wave offers a quantitative analysis for assessing the blood flow within the LAA. The images obtained by TEE are present in Figure 1.

### Statistical analysis

The statistical analysis was performed using SPSS 27.0 (SPSS, Inc.) and the R statistics package. Continuous variables exhibiting normal or near-normal distributions are represented as X ± SD, and categorical variables are presented as frequency or percentage. Calculate the 95% confidence interval (CI) according to the binomial distribution. With TEE as the reference standard, the diagnostic performance of Pulmonary venous and three delayed phases in the diagnosis of LAA thrombus was calculated by the 4-grid table method. All DLP (mGy × cm) examined were recorded and the effective radiation dose (mSv) for each phase was calculated. Independent sample t-test was used to compare the effective radiation of patients with or without thrombosis and the degree of esophageal filling and image quality between the water-swallowing group and the calm-breathing group. *p*<0.05 is considered statistically significant.

## Result

The clinical characteristics of the study population are summarized in Table 1. A total of 278 consecutive patients were enrolled in the study, including 181 males (65%), with an average age of 56 ± 13 years, ranging from 32 to 81 years. All patients underwent CT examination of the left atrium and pulmonary veins and TEE without complications. All patients were in AF during the CT scan. In all cases, the image quality is technically sufficient for clinical evaluation. Of the 278 patients, 14 (5, 95% CI 2–8%) were diagnosed with thrombus by TEE, and all of the thrombi were located in the LAA.

**Table 1:** Study characteristics of the overall populations.

Variable	Values (*n* = 278)
Age (years), mean ± SD (range)	56 ± 13 (32–81)
Males, *n* (%)	181 (65)
Body mass index (kg/m^2^), mean ± SD (range)	26.3 ± 8.3 (16.9–48.6)
Hypertension, *n* (%)	121 (43)
Diabetes mellitus, *n* (%)	25 (8)
Hyperlipidemia, *n* (%)	86 (31)
Ejection fraction ≤ 55%, *n* (%)	28 (10)
Previous TIA/stroke, *n* (%)	8 (3)
Mean CHADS_2_, mean ± SD (range)	0.8 ± 0.4 (0–3)

BMI, body mass index; TIA, Transient ischemic attack.

### Diagnostic accuracy with different delay times

**Figure 2:**
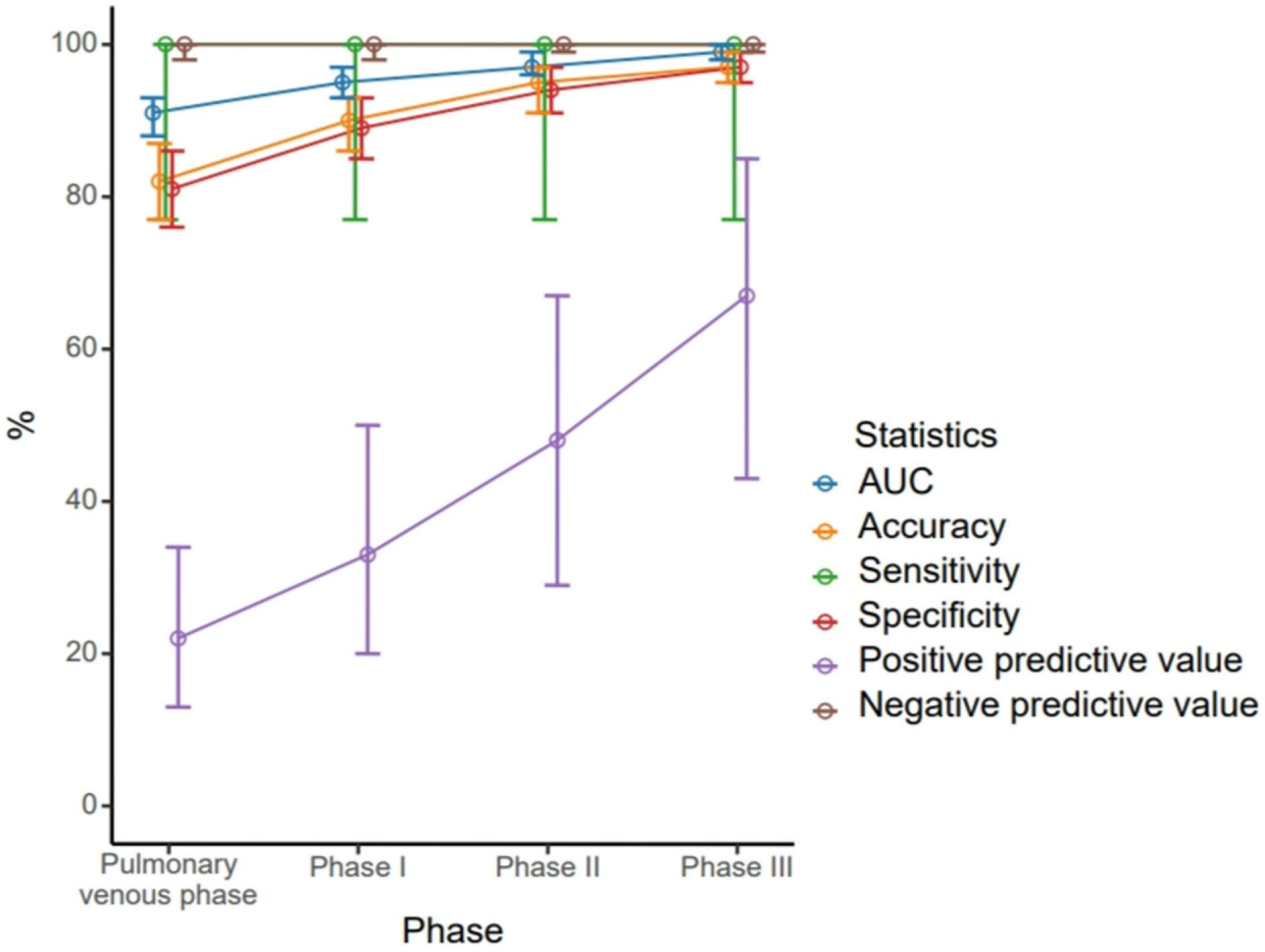
Different diagnostic efficiency of the pulmonary venous phase, phase I delayed, phase II delayed and phase III delayed for the left atrial thrombus detection.

A total of 63 patients (22.6%) had filling defects in the pulmonary vein phase, of which 42 patients (15.1%) had filling defects after the delayed phase I, 29 patients (10%) had filling defects after delayed phase II, and 21 patients (7.5%) still had filling defects after delayed phase III (Table 2). With the patient’s TEE as a reference standard, the sensitivity and negative prediction of all phases were 100%, and the specificities were 0.81, 0.89, 0.94, and 0.97, respectively; and the PPVs were 0.22, 0.33, 0.48, and 0.67, respectively; and the areas under the curve (AUC) were 0.91, 0.95, 0.97, and 0.99, respectively (Table 3). Their changes are presented in Figure 2. The diagnostic accuracy of the pulmonary venous phase and three-phase delay exhibited significant statistical differences (*p* < 0.0001).

**Table 2:** Confusion matrix in the diagnosis of LAA thrombus by CT using TEE as the gold standard.

CT		Pulmonary venous phase		Phase I delayed		Phase II delayed		Phase III delayed		Phase III delayed iodide quantification	
		Thrombus	No thrombus	Thrombus	No thrombus	Thrombus	No thrombus	Thrombus	No thrombus	Thrombus	No thrombus
TEE	Thrombus	14	0	14	0	14	0	14	0	14	0
	No thrombus	49	215	28	236	15	249	7	257	0	264

**Table 3:** Diagnostic performance of the pulmonary venous phase, phase I delayed, phase II delayed, phase III delayed and phase III delayed iodide quantification for LAA thrombus detection.

	AUC	Accuracy	Sensitivity	Specificity	PPV	Negative predictive value
Pulmonary venous phase	91 (88–93)	82 (77–87)	100 (77–100)	81 (76–86)	22 (13–34)	100 (98–100)
Phase I delayed	95 (93–97)	90 (86–93)	100 (77–100)	89 (85–93)	33 (20–50)	100 (98–100)
Phase II delayed	97 (96–99)	95 (91–97)	100 (77–100)	94 (91–97)	48 (29–67)	100 (99–100)
Phase III delayed	99 (98–100)	97 (95–99)	100 (77–100)	97 (95–99)	67 (43–85)	100 (99–100)
Phase III delayed iodide quantification	100 (100–100)	100 (99–100)	100 (77–100)	100 (99–100)	100 (77–100)	100 (99–100)

Data are expressed as value (%) and 95% confidence interval.

AUC, Area under curve; PPV, positive predictive value; NPV, negative predictive value.

### Spectral iodine concentration for diagnosis of thrombus

**Figure 3:**
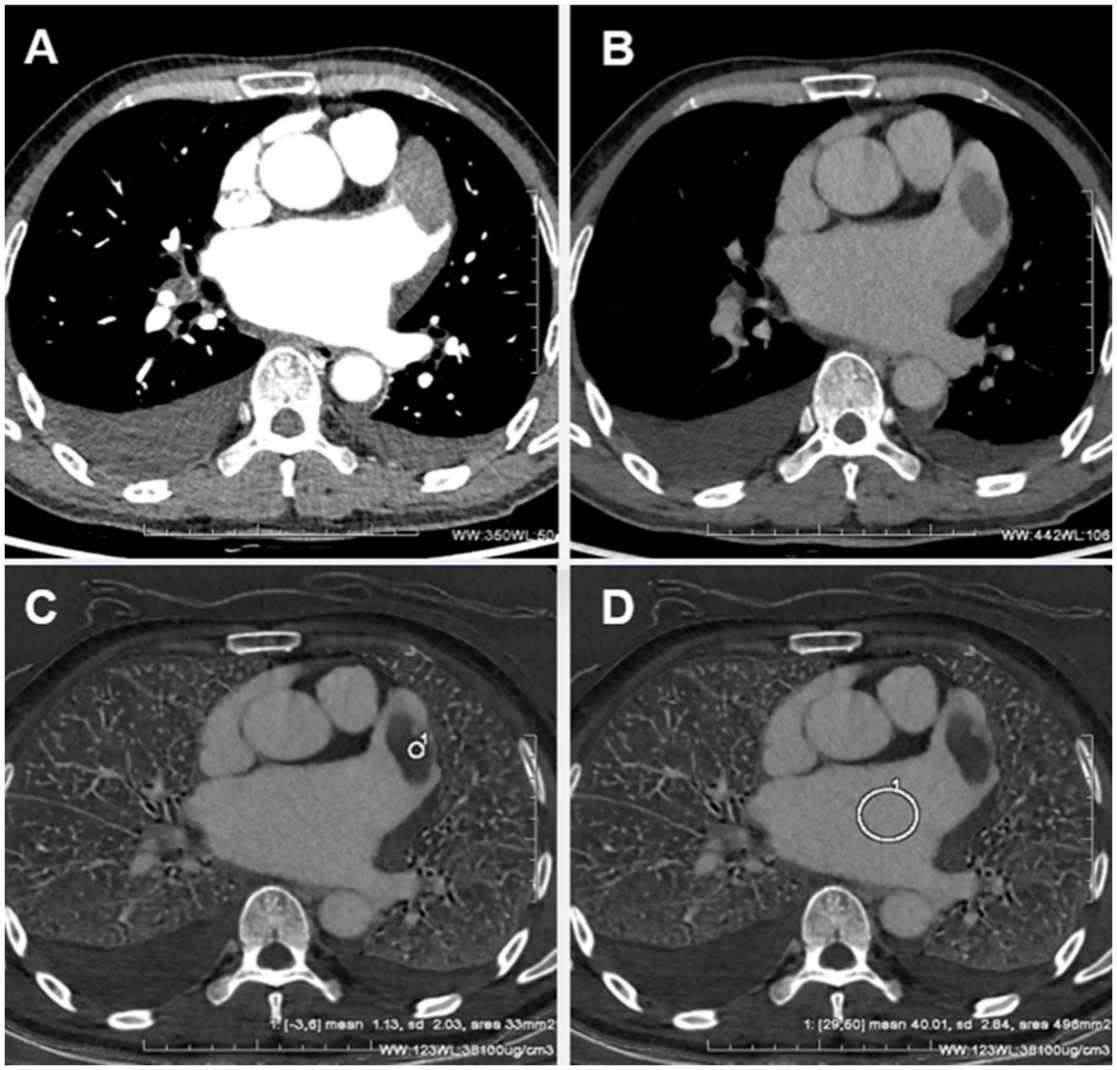
**(A)** The pulmonary venous phase shows significant filling defects in the LAA. **(B)** The delayed phase III shows the filling defect in the LAA was reduced. **(C)** The iodine concentration of the LAA filling defects was measured as 1.13 ± 2.03 ug/cm^3^ in the delayed phase III spectral iodine map. **(D)** The iodine concentration of the left atrium was measured as 40.01 ± 2.84 ug/cm^3^ in the delayed phase III spectral iodine map. The iodine concentration in the left atrium was significantly higher compared to that observed in the filling defect of the LAA. The iodine concentration in the filling defect of the LAA was nearly negligible, further substantiating it was a thrombus.

**Figure 4:**
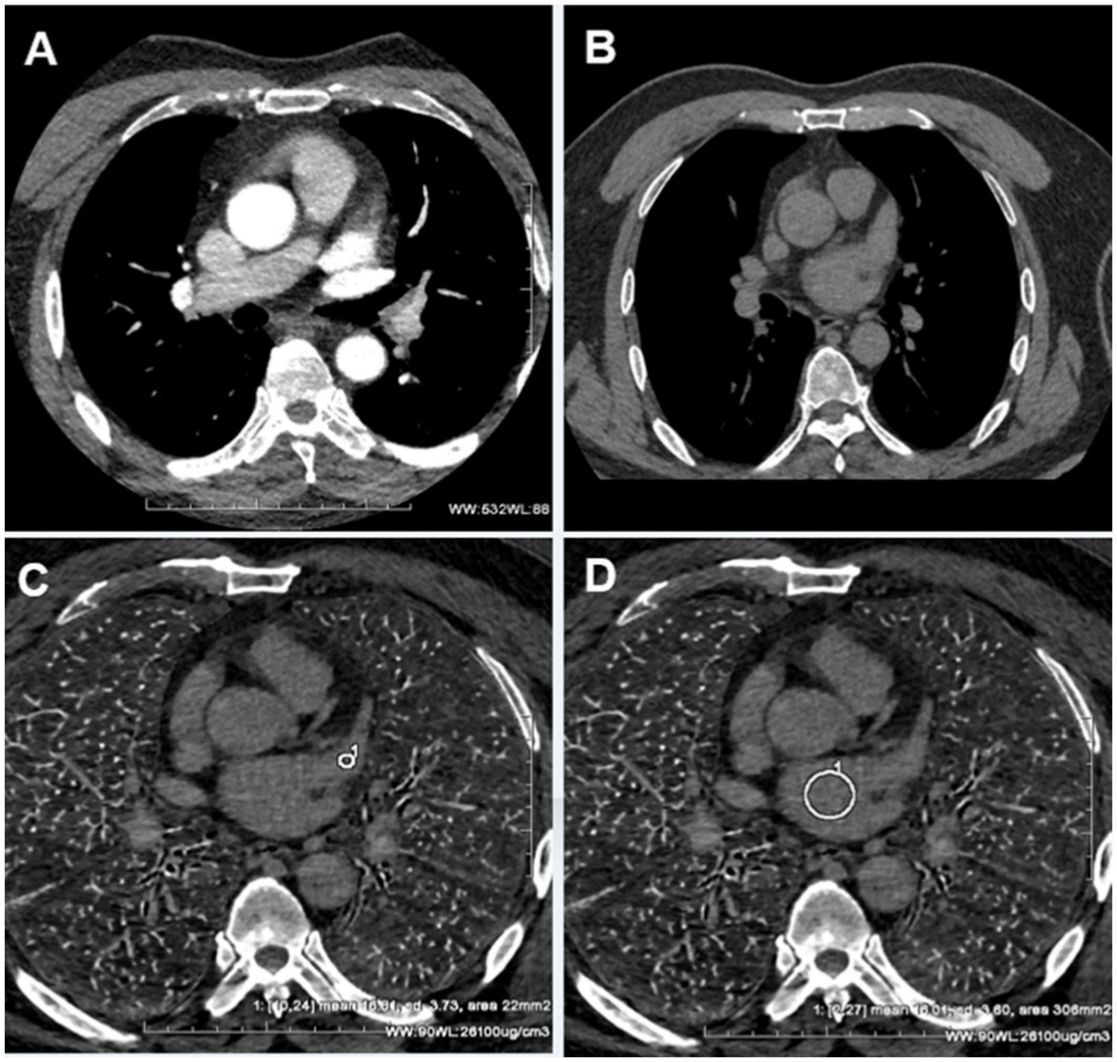
**(A)** The pulmonary venous phase shows significant filling defects in the LAA. **(B)** The delayed phase III shows the filling defect disappeared. Due to the long-term delayed loss of the iodine contrast agent, it was difficult to determine whether the LAA was filled or not. **(C)** The iodine concentration of the former LAA filling defects was measured as 16.81 ± 3.73 ug/cm^3^ in the delayed phase III spectral iodin map. **(D)** The iodine concentration of the left atrium was measured as 16.01 ± 3.01 ug/cm^3^ in the delayed phase III spectral iodine map. The iodine concentration in both the left atrium and former LAA filling defects was found to be equivalent, indicating a uniform distribution of low-concentration iodine contrast agents within these structures at this moment. Consequently, the presence of left atrial appendage thrombus can be completely ruled out.

**Figure 5:**
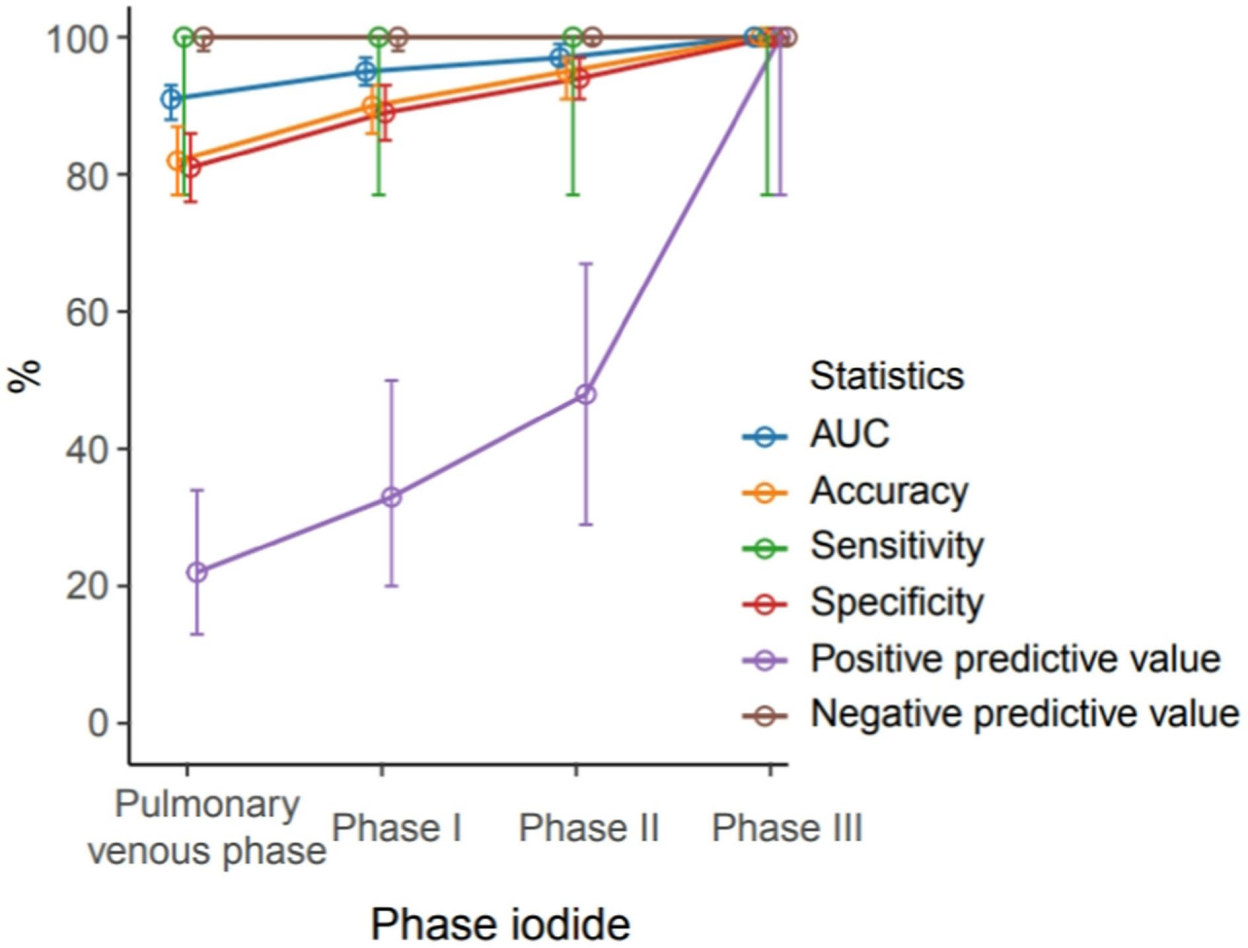
Different diagnostic efficiency of the pulmonary venous phase, phase I delayed, phase II delayed and phase III delayed combined with quantification of iodine-based substances for the left atrial thrombus detection.

During delayed phase III, routine CT detected 14 cases of filling defects, and the other 7 cases could not be determined whether filling defects or not, due to the long delay time. The loss of iodine contrast agent led to the inability to accurately judge by the naked eye (Figures 3, 4). For all delayed phase III images, the iodine concentration was quantitatively measured, with an average value of 1.36 ± 2.23 μg/cm^3^ in the LAA diagnosed with filling defect by TEE, which significantly differed from the average value of 32.21 ± 10.23 μg/cm^3^ in the left atrium (*p* < 0.001). The average value of iodine concentration in the LAA diagnosed by TEE without filling defect was 11.83 ± 4.53 μg/cm^3^, which was not significantly different from the average value of 12.32 ± 2.46 μg/cm^3^ in the left atrium (*p* > 0.05). The sensitivity, specificity, NPV, PPV, accuracy, and AUC of delayed phase III combined with iodine concentration for diagnosis of the LAA were all 100% (Table 3). Compared with the delayed phase III diagnosis alone, the diagnostic efficacy of thrombosis combined with spectral iodine concentration was significantly improved, as shown in Figure 5.

### Radiation dosage

The average radiation dose in the pulmonary venous phase was 2.38 ± 1.10 mSv (1.54–5.78 mSv), and the delayed radiation doses in the subsequent three phases were 0.334 ± 0.142 mSv (0.22–1.12 mSv), 0.325 ± 0.140 mSv (0.21–1.08 mSv), and 0.334 ± 0.139 mSv (0.22–1.11 mSv), respectively. For all patients, the average radiation dose of the complete CT examinations was 3.377 ± 1.534 mSv (2.21–6.08 mSv), while the maximum radiation dose for patients receiving all delayed-phase scans (*n* = 63, 22%) was 5.11 ± 1.05 (1.78–6.08) mSv.

### Esophageal dilation and image quality evaluation

In the water-swallowing group, 18 cases exhibited complete esophageal filling, 6 cases showed 2/3 length filled, 4 cases had 1/2 length filled, and 2 cases had a complete closed contraction (primarily due to inadequate patient cooperation). In contrast, the calm-breathing group all displayed closed contractions. The subjective score of image quality in the water-swallowing group was 4.6, whereas 4.5 in the calm-breathing group. There was no statistically significant difference observed in the subjective scores between the two groups (*p* > 0.05). The SNR and CNR of the water-swallowing group and the calm-breathing group were 30.365 ± 11.508, 27.895 ± 10.191 and 30.267 ± 13.248, 28.325 ± 11.362, respectively, with no statistically significant differences observed between the two groups (*p* > 0.05). In the water-swallowing group, the age stratification for esophagus expansion examine is shown in Table 4, with the elderly (>60 years) accounting for half. Among patients with full esophageal dilation, the elderly account for half of the middle-aged population, while complete closure only occurs in the elderly.

**Table 4:** Esophageal dilation in elderly individuals (>60 years) and adults (<60 years) in the water-swallowing group.

Esophageal dilation		Complete filling	2/3 length filled	1/2 length filled	Complete closed
Age	>60 year (*N* = 15)	12	2	1	0
	<60 year (*N* = 15)	6	4	3	2

## Discussion

Radiofrequency ablation and PVI surgery are required for patients with AF. The surgeon is extremely concerned with the anatomical relationship between the left atrium, pulmonary vein, and esophagus, besides the presence of a thrombus in the LAA (15–17). The location of the esophagus exhibits significant variability, though the precise anatomical position is crucial for radiofrequency ablation procedures. If the esophagus obstructs the pulmonary vein isolation site, physicians must judiciously adjust or minimize the application of radiofrequency energy to prevent potential complications, such as thermal injury leading to esophageal fistula formation (18, 19). Most hospital radiology departments are capable of providing high-quality three-dimensional reconstruction images of the left atrium and pulmonary veins, as facilitated by iodinated contrast agents and enhanced CT. However, because of the cavity structure, the esophagus often poses challenges for accurate three-dimensional reconstructing. In this study, we combined CT protocols to adjust the esophageal threshold, enabling simultaneous visualization of the high-density left atrium, pulmonary vein, and low-density esophagus in a three-dimensional manner.

**Figure 6:**
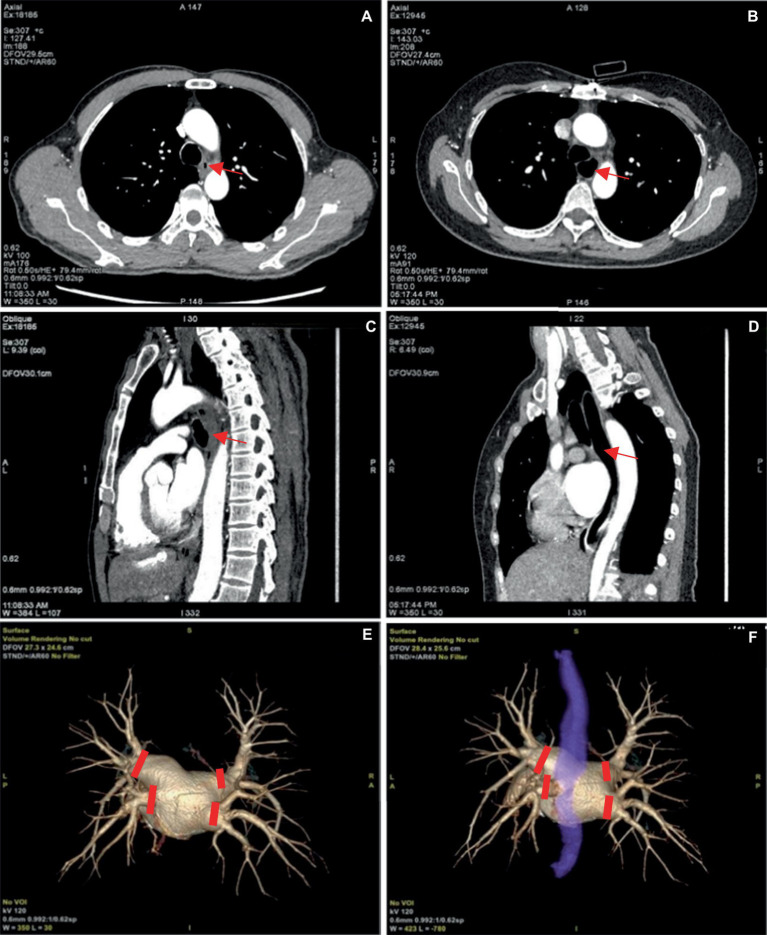
**(A,C)** Represent the pulmonary venous phase axial and sagittal images of the same patient, with calm breathing during image acquisition. And the esophagus is seen in a contracted state at the arrow. **(B,D)** Depict the pulmonary venous phase axial and sagittal images of the same patient with water-swallowing during image acquisition. And well-filled and expanded esophagus is indicated at the arrow. **(E)** Shows the three-dimensional reconstructed image of the pulmonary vein. **(F)** Depicts the fusion image of the left atrium, pulmonary vein, and esophagus in a water-swallowed patient, with an esophagus stained blue. The four red markers indicate the points for pulmonary vein isolation during radiofrequency ablation for atrial fibrillation.

Our attempts enabled convenient visualization of the anatomical relation of the esophagus (blue, Figure 6). The esophagus is in a constricted state during non-feeding conditions, leading to indistinct of the boundary between the esophageal wall and the surrounding soft tissue during reconstruction, and low CNR and SNR of the image. Expanding the esophagus during image acquisition may facilitate image reconstruction. For this purpose, the patients were asked to wrap a mouthful of diluted iodine contrast agent in the mouth and follow the voice instructions to swallow during image acquisition. This approach significantly enhanced esophageal expansion, however, the high density of the diluted iodine contrast agent resulted in partial sclerosis artifacts surrounding the esophagus and we excluded this method. Subsequently, we employed water-swallowing, which effectively dilated the esophagus without compromising the SNR. For radiofrequency ablation surgeons, the three-dimensional fusion images in the water-swallowing group exhibited significantly superior quality compared to those in the calm-breathing group. However, it is more difficult for elderly people to undergo swallowing tests. For example, in our study, there was an 80-year-old man who did not respond to the swallowing instruction during the examination. Though his esophageal dilation was not perfect, we included the case in the study because it reflects the current needs.

Several studies have demonstrated that cardiac CT owns an NPV of 100% for detecting LAA thrombus. The incorporation of delayed enhancement can significantly improve the specificity to 98.1% and the PPV to 95.76% (5–10, 20). Particularly, Spagnolo et al. established various delay times based on their professional expertise and experience, ranging from 1 to 6 min, while Li et al. employed delay times of 1, 2, and 3 min (9, 10). In our study, we optimized the first delay time to 30 s. In our result, 21 cases had the filling defects of the LAA disappeared, suggesting that a large proportion of cases may not need to be delayed for too long. To this end, we set the first delay time to 30 s, saving examination time and optimizing the process.

Most studies of CT examination used delayed enhancement to diagnose the LAA, and only a few studies mentioned applied iodine concentrations, with comparatively lower diagnostic accuracy (20–24). To be noted, this multimodal diagnosis combines with delay enhancement and spectral iodine concentration quantification. Especially in delayed phase III, the delay time amounts to 30 s plus 1 min plus 2 min, which was relatively long, and the iodine contrast agent content in the left atrium and LAA continued to decline, so it was hard to determine whether the iodine contrast agent had been lost or filled with low concentration evenly by the naked eye. Therefore, we measured the iodine concentration of the LAA and left atrium at the same level for quantitative analysis of the iodine contrast agent, for further determination of whether there is a thrombus in the LAA.

CMR is one of the favorable diagnostic techniques for detecting and evaluating left atrial and left atrial thrombosis, with the advantage of avoiding ionizing radiation and iodinated contrast agents. As a technique for excluding left ventricular thrombus, however, CMR used for LAA thrombus detection is less common. Tasnim et al. (25) conducted a meta-regression analysis comparing the efficacy of CMR and TEE in diagnosing LAA thrombi, and found that the sensitivity of CMR was 0.80 (CI 0.63–0.91) and the specificity was 0.98 (CI 0.07–0.99). It is noteworthy that Kitkungvan et al. suggest that equilibrium phase delayed enhancement (DE) CMR with a long inversion time (TI) of 600 ms (long TI DE-CMR) had a diagnostic accuracy of 99.2%, sensitivity of 100%, and specificity of 99.2% for thrombus detection (26). So far, this MR imaging technique has the highest diagnostic efficacy for LAA thrombi, which is comparable to our study. Also, Tasnim et al. (25) analyzed previous studies and found that delayed CCT had higher sensitivity and specificity than CMR with no significant difference (*p* value 0.369 and 0.828). Together, CMR plays an important role in LA and LAA imaging, several reasons such as the limited availability of scanners, claustrophobic patients, or the presence of CMR unsafe devices may restrict its use in favor of CCT.

This study together with the current domestic and international landscape, a novel preoperative examination protocol for radiofrequency ablation was proposed. We recommend utilizing CT as the primary diagnostic modality for LAA thrombus, instead of TEE. Delayed enhancement combined with spectral iodine concentration is comparable to TEE in diagnosing LAA thrombus, while CT image reconstruction offers a superior anatomical position relationship of the left atrium, pulmonary veins, and esophagus compared to TEE. Particularly during the COVID-19 pandemic, the proportion of TEE examinations at Johns Hopkins Hospital decreased from 34.6 to 3.7%, while computed tomography angiography (CTA) increased from 74.8 to 93.9% (27). The adoption of CT as an alternative diagnostic tool for LAA thrombus effectively minimized unnecessary invasive procedures and examination expenses. The heightened risk of COVID-19 transmission associated with TEE insertion via mouth was mitigated. Therefore, CT as an alternative to TEE for diagnosing LAA thrombus may establish a novel standard of examination; but current expert guidelines advocate further research for CT.

There were several limitations to this study, such as a single-center retrospective evaluation. Future research should be conducted in multiple radiation institutions and a large population to determine whether the methods are easy to achieve repeatability and standardization. Also, we did not conduct the stratified analysis of cardiac function, atrial fibrillation type, CHADS_2_ score, etc., in patients with suspected LAA thrombus. Moreover, we did not investigate the association between these indicators and delay time. In future investigations, it is conceivable to stratify high-risk patients and employ a two-minute delayed scan directly on individuals with a strong suspicion of LAA thrombus to mitigate radiation exposure. In addition, we did not investigate whether patients who did not indicate the presence of atrial thrombi on CT experienced thromboembolism after radiofrequency ablation, as this requires long-term follow-up and monitoring of postoperative patients. Also, due to equipment limitations, we did not perform CMR imaging on patients with left atrial appendage thrombosis. However, research on the comparison of CCT and CMR for left atrial imaging within the same patients with AF assessed the diagnosis efficacy for left atrial and LAA thrombi and evaluated their respective advantages in displaying left atrial and LAA.

## Conclusion

The CT examination of the left atrium and pulmonary veins combined with the delayed phases, demonstrates high accuracy in diagnosing LAA thrombus. We quantified the iodine concentration and achieved comparable diagnostic efficacy to TEE, with an overall radiation dose below 3.5 mSv. Additionally, instructing the patient to swallow purified water during the examination can facilitate optimal distension and filling of the esophagus. The resulting three-dimensional reconstruction provides a more intuitive visualization of the anatomical structure of the esophagus, left atrium, and pulmonary vein, thereby offering valuable assistance to clinicians in performing radiofrequency ablation surgery. However, future multicenter studies are warranted to substantiate the efficacy of our research methodology.

## Data Availability

The raw data supporting the conclusions of this article will be made available by the authors, without undue reservation.

## References

[1] Bizhanov KA Аbzaliyev KB Baimbetov AK Sarsenbayeva AB Lyan E. Atrial fibrillation: epidemiology, pathophysiology, and clinical complications. J Cardiovasc Electrophysiol. (2023) 34:153–65. doi: 10.1111/jce.15759 36434795

[2] Joglar JA Chung MK Armbruster AL Benjamin EJ Chyou JY Cronin EM. 2023 ACC/AHA/ACCP/HRS guideline for the diagnosis and Management of Atrial Fibrillation: a report of the American College of Cardiology/American Heart Association joint committee on clinical practice guidelines. Circulation. (2024) 149:e1–e156. doi: 10.1161/CIR.0000000000001193, PMID: 38033089 PMC11095842

[3] Chieng D Sugumar H Ling LH Segan L Azzopardi S Prabhu S. Catheter ablation for persistent atrial fibrillation: a multicenter randomized trial of pulmonary vein isolation (PVI) versus PVI with posterior left atrial wall isolation (PWI) - the CAPLA study. Am Heart J. (2022) 243:210–20. doi: 10.1016/j.ahj.2021.09.015, PMID: 34619143

[4] Buist TJ Zipes DP Elvan A. Atrial fibrillation ablation strategies and technologies: past, present, and future. Clin Res Cardiol. (2021) 110:775–88. doi: 10.1007/s00392-020-01751-5, PMID: 33089361

[5] Hur J Kim YJ Lee HJ Nam JE Ha JW Heo JH. Dual-enhanced cardiac CT for detection of LAA thrombus in patients with stroke: a prospective comparison study with TEE. Stroke. (2011) 42:2471–7. doi: 10.1161/STROKEAHA.110.611293, PMID: 21757676

[6] Romero J Cao JJ Garcia MJ Taub CC. Cardiac imaging for assessment of LAA stasis and thrombus. Nat Rev Cardiol. (2014) 11:470–80. doi: 10.1038/nrcardio.2014.77 24913058

[7] Lazoura O Ismail TF Pavitt C Lindsay A Sriharan M Rubens M. A low-dose, dual-phase cardiovascular CT protocol to assess LAA anatomy and exclude thrombus before left atrial intervention. Int J Cardiovasc Imaging. (2016) 32:347–54. doi: 10.1007/s10554-015-0776-x, PMID: 26420491

[8] Kawaji T Numamoto H Yamagami S Mabuchi R Kitamura T Enoki N. Real-time surveillance of LAA thrombus during contrast computed tomography imaging for catheter ablation: the reliability of computed tomography beyond UltraSound in THROMBUS detection (THROMBUS) study. J Thromb Thrombolysis. (2019) 47:42–50. doi: 10.1007/s11239-018-1742-y, PMID: 30251193

[9] Spagnolo P Giglio M Di Marco D Cannaò PM Agricola E Della Bella PE. Diagnosis of LAA thrombus in patients with atrial fibrillation: delayed contrast-enhanced cardiac CT. Eur Radiol. (2021) 31:1236–44. doi: 10.1007/s00330-020-07172-2, PMID: 32886202 PMC7880950

[10] Li XN Wang JX Wei Q Yu XB Zhou YT Ma XY. Diagnostic value of delayed contrast-enhanced cardiac computed tomography for detecting LAA Thrombus in patients with atrial fibrillation. Front Cardiovasc Med. (2022) 9:847163. doi: 10.3389/fcvm.2022.847163, PMID: 35571218 PMC9095922

[11] Donal E Lip GYH Galderisi M Goette A Shah D Marwan M. EACVI/EHRA expert consensus document on the role of multi-modality imaging for the evaluation of patients with atrial fibrillation. Eur Heart J Cardiovasc Imaging. (2016) 17:355–83. doi: 10.1093/ehjci/jev354, PMID: 26864186

[12] Regazzoli D Ancona F Trevisi N Guarracini F Radinovic A Oppizzi M. LAA: physiology, pathology, and role as a therapeutic target. Biomed Res Int. (2015) 2015:1–13. doi: 10.1155/2015/205013, PMID: 26236716 PMC4508372

[13] Kwon H Cho J Oh J Kim D Cho J Kim S. The adaptive statistical iterative reconstruction-V technique for radiation dose reduction in abdominal CT: comparison with the adaptive statistical iterative reconstruction technique. Br J Radiol. (2016) 88:20150463. doi: 10.1259/bjr.20150463 PMC4730981 26234823

[14] McCollough CH Primak AN Braun N Kofler J Yu L Christner J. Strategies for reducing radiation dose in CT. Radiol Clin North Am. (2009) 47:27–40. doi: 10.1016/j.rcl.2008.10.006, PMID: 19195532 PMC2743386

[15] Kumar S Brown G Sutherland F Morgan J Andrews D Ling LH. The transesophageal echo probe may contribute to esophageal injury after catheter ablation for paroxysmal atrial fibrillation under general anesthesia: a preliminary observation. J Cardiovasc Electrophysiol. (2015) 26:119–26. doi: 10.1111/jce.12575, PMID: 25352207

[16] McLellan AJ Ling LH Ruggiero D. Pulmonary vein isolation: the impact of pulmonary venous anatomy on long-term outcome of catheter ablation for paroxysmal atrial fibrillation. Heart Rhythm. (2014) 11:549–56. doi: 10.1016/j.hrthm.2013.12.025, PMID: 24342795

[17] Qi D Zhang J. Relationship between anatomical characteristics of pulmonary veins and atrial fibrillation recurrence after radiofrequency catheter ablation: a systematic review and meta-analysis. Front Cardiovasc Med. (2023) 10:1235433. doi: 10.3389/fcvm.2023.1235433, PMID: 37795484 PMC10546190

[18] Orosey M Garg L Agrawal S John JJ Haines DE Wong WS. Atrioesophageal fistula following radiofrequency catheter ablation of atrial fibrillation. Rev Cardiovasc Med. (2017) 18:115–22. doi: 10.3909/ricm0883 29111544

[19] Back Sternick E Soares Correa F Ferber Drumond L Albuquerque Carreiro R Alves Rabelo M Tarso Vaz de Oliveira P. Esophago-pericardial fistula after catheter ablation of atrial fibrillation: a review. J Cardiovasc Electrophysiol (2020) 31: 2600–2606, doi: 10.1111/jce.14723, PMID: 32829527

[20] Guha A Dunleavy MP Hayes S Afzal MR Daoud EG Raman SV. Accuracy of contrast-enhanced computed tomography for thrombus detection before atrial fibrillation ablation and role of novel LAA enhancement index in appendage flow assessment. Int J Cardiol. (2020) 318:147–52. doi: 10.1016/j.ijcard.2020.06.035, PMID: 32629004

[21] Zeng H Zhang MC He YQ Liu L Tong YL Yang P. Application of spectral computed tomography dual-substance separation technology for diagnosing left ventricular thrombus. J Int Med Res. (2016) 44:54–66. doi: 10.1177/0300060515600186 26658269 PMC5536565

[22] Li W Yu F Zhu W Zhang W Jiang T. Detection of LAA thrombi by third-generation dual-source dual-energy CT: iodine concentration versus conventional enhancement measurements. Int J Cardiol. (2019) 292:265–70. doi: 10.1016/j.ijcard.2019.04.079 31072634

[23] Schlett CL Heidt MC Jörg A Soschynski M Buss SJ Korosoglou G. Mehrwert der dual-energy-Computertomographie zur Detektion von Thromben des linken Vorhofohrs [value of dual-energy computed tomography for detection of LAA thrombus]. Radiologe. (2020) 60:1162–8. doi: 10.1007/s00117-020-00774-3, PMID: 33237385

[24] Hong YJ Hur J Han K Im DJ Suh YJ Lee HJ. Quantitative analysis of a whole cardiac mass using dual-energy computed tomography: comparison with conventional computed tomography and magnetic resonance imaging. Sci Rep. (2018) 8:15334. doi: 10.1038/s41598-018-33635-0, PMID: 30337716 PMC6194136

[25] Vira T Pechlivanoglou P Connelly K Wijeysundera HC Roifman I. Cardiac computed tomography and magnetic resonance imaging vs. transoesophageal echocardiography for diagnosing left atrial appendage thrombi. Europace (2019) 21: e1–e10.doi: 10.1093/europace/euy142. 29961869 10.1093/europace/euy142

[26] Kitkungvan D Nabi F Ghosn MG Dave AS Quinones M Zoghbi WA. Detection of LA and LAA Thrombus by CMR in patients referred for pulmonary vein isolation. JACC Cardiovasc Imaging. (2016) 9:809–18. doi: 10.1016/j.jcmg.2015.11.029, PMID: 27236529

[27] Akhtar T Wallace R Daimee UA Hart E Arbab-Zadeh A Marine JE. Transition from TEE to cardiac computed tomography for the evaluation of LAA thrombus before atrial fibrillation ablation and incidence of cerebrovascular events during the COVID-19 pandemic. J Cardiovasc Electrophysiol. (2021) 32:3125–34. doi: 10.1111/jce.15227, PMID: 34453377

